# Herpes simplex virus detection and genomes from under-sampled, remote populations

**DOI:** 10.1371/journal.pone.0344138

**Published:** 2026-07-24

**Authors:** Christopher D. Bowen, Alexandre Blake, Daniel W. Renner, Mary Ashley Hazel, John Jakurama, Justy Matundu, Moriah L. Szpara, Nita Bharti

**Affiliations:** 1 Department of Biology, The Center for Infectious Disease Dynamics, Huck Institutes for the Life Sciences, Pennsylvania State University, University Park, Pennsylvania, United States of America; 2 Department of Biochemistry and Molecular Biology, Pennsylvania State University, University Park, Pennsylvania, United States of America; 3 Institute for Health & Aging, School of Nursing, University of California, San Francisco, California, United States of America; 4 Kaoko Information Centre, Opuwo, Namibia; Albert Einstein College of Medicine, UNITED STATES OF AMERICA

## Abstract

Herpes simplex virus (HSV) is an endemic pathogen, infecting over half of all adults world-wide. HSV infection can cause a wide spectrum of disease outcomes, ranging from asymptomatic infection or mild lesions to rare cases of infectious keratitis, encephalitis, and death. HSV genome sequences differ between individuals and within individuals. To date, the vast majority of publicly available HSV genomic data has come from Europe and North America. Populations in South America, Africa, and Asia are under-sampled, as are non-industrial (e.g., agricultural, pastoral) populations, for which the natural environment plays a large role in health and disease dynamics. We used Whatman FTA card stabilization of DNA to develop a procedure for capturing oral and genital swabs from a geographically isolated pastoralist population in a desert region of northern Namibia. This is the first study to document HSV genome sequences from this type of remote setting and these are the first HSV genomes from Namibia. The resulting HSV sequences, collected in 2015 and 2016 from remote settlements in Namibia, fit within the scope of viral genetic diversity previously defined by African strains. The methodological approaches developed in this study can be expanded to broaden viral detection, improve diagnostics, and raise public health awareness about the burden of pathogens in under-served populations.

## Introduction

Herpes simplex viruses (HSV) are large double-stranded DNA (dsDNA) viruses classified in the *Alphaherpesvirinae* sub-family of the *Herpesviridae* family [1]. There are two distantly related species, HSV-1 and HSV-2 (*Simplexvirus humanalpha1 or 2*), that infect human oral or genital niches. HSV-1 has established lifelong infections in about 67% of adults worldwide, or an estimated 3.7 billion people under the age of 50, while HSV-2 has infected an estimated 491 million [2]. While primary infection by HSV occurs at the mucosal epithelium, lifelong latency is established in the nervous system [1]. From this neuronal reservoir, the virus can reactivate in response to stressful stimuli. Reactivation enables the virus to return to the skin surface, initiate new shedding and/or lesions, and potentially transmit to new hosts.

Previous studies have shown that HSV isolates from unrelated individuals can differ between 2−4% in DNA identity genome-wide [3,4]. HSV-1 co-evolved with human ancestors while HSV-2 more recently crossed into humans and exhibits less diversity between human isolates [4–6]. Both HSV species have large dsDNA genomes of over 150 kb, which encode >75 proteins. This amount of variation can produce distinct coding differences in >60 proteins between any two unrelated isolates of HSV-1 [3,4,7]. HSV acquires genetic diversity by a variety of mechanisms, notably fluctuation in tandem repeat (TR) lengths, insertions/deletions (in/dels), single nucleotide polymorphisms (SNPs), and recombination events. Isolates with common geographic origins tend to group together in phylogenetic analyses, though these groupings show intermingling with the addition of recent isolates [3,4,6–15].

To date, the vast majority of HSV-1 isolates sequenced have been collected from urban clinics in either North America or Europe [3,7,11–15]. In contrast, multiple studies have characterized HSV-2 genomes from not only North America and Europe, but also Peru, South Africa, Zimbabwe, Zamiba, Uganda, Kenya, and Tanzania [16–21]. However, there remains a significant underrepresentation of HSV samples from South America, Africa, and Asia as well as from subsistence populations and rural areas globally [8–10,22,23]. Geographically isolated populations are difficult to sample, and the long-term chronic nature of HSV infections may lead to tolerance of symptoms, dissuading individuals from seeking formal diagnoses or ongoing treatment, which increases the difficulty of sample collection. It is crucial to understand the genetic diversity of HSV circulating in humans globally to develop broadly effective and equitable diagnostics and treatments.

In this study, we quantified HSV shedding in a pastoralist population in Namibia through two data collection efforts, conducted during the rainy season of 2015 and the dry season of 2016. We focused on the Kaokoveld region of northwestern Namibia. The area is a sparsely populated desert, with nearly all participants self-identifying as members of the Himba tribe. A small number of participants identified with additional tribes residing in the area, including the Tjimba, Ovambo, Zemba, and Twe [24–26]. These groups are Bantu-speaking with cultural ties to semi-nomadic pastoralism, supported by subsistence agriculture. In the typical gender-based division of labor in Kaokoveld, men primarily tend cattle herds, which requires frequent dispersal from home villages to find grazing and water. Women manage subsistence farms, goat herds, and childcare. Although men are more mobile than women, it is not uncommon for residents of this region to be away from their primary residence for long periods of time (weeks or months), either for subsistence or to visit family. Sexual partner concurrency is common and tolerated for men and women and occurs through extra-marital relationships and polygynous marriage.

The work presented here is part of a larger study about community health in a remote population, including access to health care, contact networks, and movement in pastoralist regions of Namibia [24–27]. Prior studies in this population have focused on sexual health and quantified the overall high seroprevalence of HSV-2 and incidence of other sexually transmitted infections; HSV-1 prevalence in this region is unknown, as are many other indicators of health [24–27]. We invited enrollment in the study regardless of exposure to HSV or symptoms of HSV. We interviewed participants about their interpersonal contacts, access to healthcare, movement, and basic demographic information. Participants also provided self-collected swabs of oral and genital areas. These samples were analyzed for HSV DNA detection. For samples with sufficient DNA, we completed viral genome sequencing and comparative genomic analysis. We paired viral DNA detection with contact-network analysis to integrate data on HSV shedding with human interaction networks. Although detectable HSV shedding was sparse and appeared seasonally variable, these approaches demonstrate proof of principle that viral sampling and storage is feasible in remote settings and illustrate the potential to integrate data on human and viral interaction networks.

## Results

### Data collection from remote settlements in Kaokoveld, Namibia

**Fig 1:**
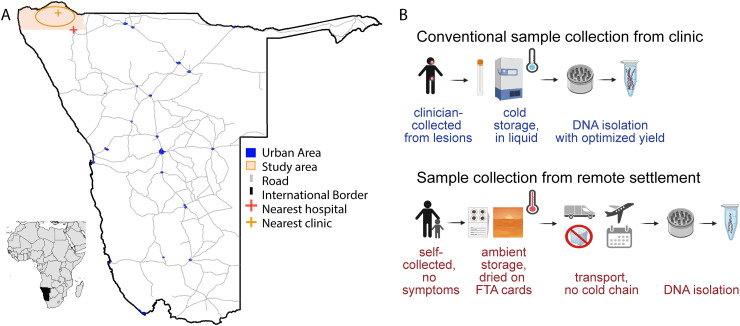
Map of study area and collection of viral DNA. (A) Lower left inset: Namibia shaded in black. Enlarged map: Urban areas in blue, approximate location of study areas in orange oval in the north. Location of nearest clinic shown with orange cross, nearest hospital shown with a red cross. Major roads in grey lines (from World Bank). Maps were created in ArcGIS and Adobe Illustrator based on modified shapefiles from World Bank and Global Rural Urban Mapping Extents. (B) Top row: conventional clinical HSV sample collection from lesions; bottom row: sample collection from asymptomatic individuals in remote settlements, as executed for this study. Diagram components were created in BioRender.

We collected biological samples and completed detailed interviews in the Kaokoveld region of northwestern Namibia (Fig 1A). These efforts spanned two site visits, one in February 2015 that occurred during a long period of drought, and the second in October 2016 that followed a period of adequate rainfall. Participants were enrolled in the study regardless of HSV symptoms or exposure.

Study participation for adults included a long-form interview and self-collection of oral and genital samples for detection of viral DNA. Biological sampling and data collection was less comprehensive for juvenile participants. For each juvenile participant across both years of data collection, an adult guardian collected a single internal oral swab (buccal) and provided basic demographic and familial information (29 in 2015, 28 in 2016; details below). In 2015, for 75 adults, we completed full length interviews (37 male [M] and 38 female [F]) and collected biological samples (details below). (During a pilot phase of the study, we collected an additional 25 biological samples (12 M, 13 F) from adults paired with basic demographic data and oral swabs only with basic demographic data from an additional two adults (2 F), details in Materials and Methods). Of these individuals, 1 M and 1 F reported experiencing genital lesions in the previous 12 months that may have been consistent with HSV infection, while 15 M and 8 F reported experiencing potential HSV oral lesions in their lifetime. In 2016, a total of 65 adults completed interviews and provided samples (31 M, 34 F). Of the 2016 study participants, 0 M and 3 F reported experiencing potential genital lesions in the previous 12 months, and 21 M and 5 F reported experiencing potential HSV oral lesions in their lifetime. Eight adults were interviewed and provided samples in both 2015 and 2016. Across both years, zero study participants reported lesions at the time of the swab collection; HSV DNA detected in this study likely reflects asymptomatic shedding.

**Fig 2:**
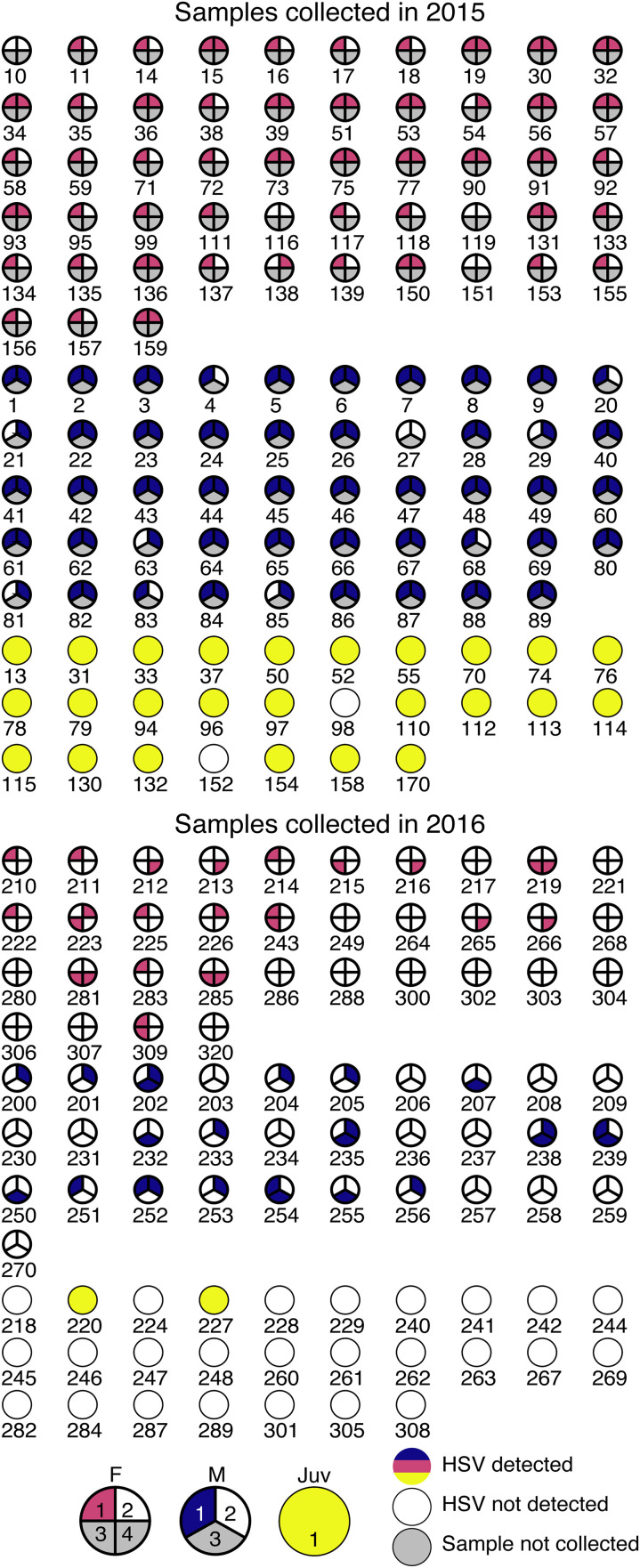
HSV detection in participant samples. Samples were numbered from 1 to 4 as follows: 1 = oral external; 2 = genital external; 3 = oral internal; 4 = genital internal. Each circle represents an individual, the sample locations from the individual are shown in clockwise sections around each circle, and colors indicate HSV detection by sample source (females in red, males in blue, juveniles in yellow) or HSV not detected (white) and sample not collected (grey). Samples 1 and 2 were collected from all adults across all years, sample 3 was collected in 2016 only from all adults, sample 4 was collected in 2016 only from all adult female participants. A maximum of two oral samples (1, oral external, and 3, oral internal) and two genital (2, genital external, and 4, genital internal) samples were taken from female adults, and their plots are divided into corresponding quarters. A maximum of two oral samples (1, oral external, and 3, oral internal) and one genital sample (2, genital external) were taken from male adults, and their plots are divided into corresponding thirds. Only one sample was taken from juveniles (1, oral cheek swab), so these plots are not divided.

We developed a low-technology approach to store and transport biological samples for later HSV detection and DNA isolation; samples needed to be compact and lightweight and could not require liquid medium, cold-storage, or electricity and could not be transported with potentially live viruses (Fig 1B). We used the following procedure: Participants’ swabs were immediately saturated in preservative-free saline and applied to Whatman FTA cards [28]. These FTA cards trap nucleic acids present in the sample, inactivating potential pathogens. They are ideal for safe handling, ambient storage, and transport. During 2015 and 2016, adult participants self-collected samples from their external oral (lip) and genital areas (labia majora, shaft of penis) (see Materials and Methods for details, and Fig 2 for overview of samples). In 2016, we added the self-collection of internal oral (buccal) swabs for males and females, and internal genital swabs for females (labia minora). A total of 486 samples were collected, with n = 229 from 2015, and n = 257 from 2016 (Fig 2).

**Fig 3:**
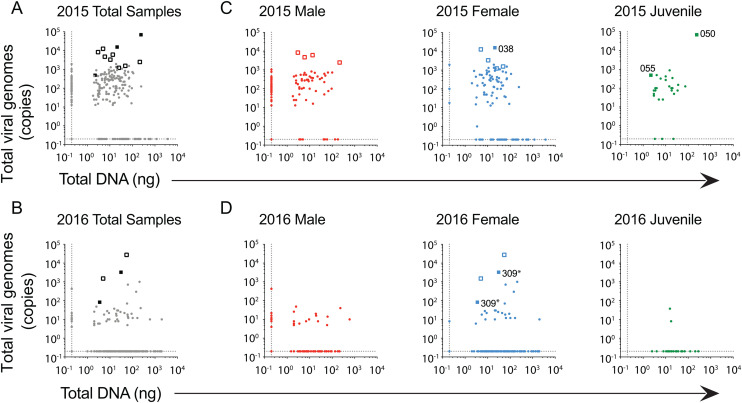
Quantification of total DNA and viral-specific DNA extracted from participant samples. Total DNA (in ng) isolated from FTA cards (x-axis) was compared to the total amount of virus-specific DNA (y-axis; viral genome copy number). Total DNA was quantified by fluorimetry and HSV-specific DNA was quantified by qPCR (see Methods for details). Data are shown for samples from the (A) 2015 data collection and (B) 2016 data collection. Samples for which viral genome sequencing was attempted are shown as hollow boxes (2015 male: 061−2, 007−2, 006−1, 004−1; 2015 female: 019−1, 032−2, 077−1, 017−1; 2016 female: 210−1, 212−4). Solid bold boxes indicate successfully sequenced samples (2015 female: 038; 2015 juveniles: 050, 055; 2016 female: 309 combined). Source labels for the successfully sequenced viral samples are indicated on specific points in (C,D). Subsets of these data are plotted separately for (C,D) male, female, and juvenile samples for each study year. The 2015 study included 229 samples: 98 swabs from 49 males; 102 swabs from 51 females; and 29 swabs from juveniles. The 2016 study included 257 samples: 93 swabs from 31 males; 136 swabs from 34 females; and 28 samples from juveniles. Two samples (oral-external and genital-external) from study participant 309 were pooled to obtain sufficient viral DNA for oligo-enrichment and sequencing.

We used quantitative PCR (qPCR) for HSV DNA to explore the potential for detection of asymptomatic viral shedding. After DNA isolation from the Whatman FTA cards, we quantified the amount of total DNA and the copy number of HSV genomes in each sample (by Qubit and qPCR respectively; see Materials and Methods for details). A total of 233 of the 486 samples analyzed (48%) had detectable HSV DNA. From these, we detected >1,000 viral genomes in only 22 samples (4.5%) (Table 1). The total sample pool (n = 229) collected in 2015 included 98 samples from 49 males; 102 samples from 51 female participants; and 29 samples from 29 juvenile participants (Fig 2). Of these samples collected in 2015, we detected >1,000 viral genomes in 18 samples (Fig 3A,C). From 2016, the sample pool (n = 257) included 93 samples from 31 male participants; 136 samples from 34 female participants; and 28 samples from 28 juvenile participants (Fig 2). Of these samples collected in 2016, we detected >1,000 viral genomes in just 4 samples (Fig 3B,D). There was no apparent correlation between the total amount of DNA and the number of HSV genomes detected in each sample (Fig 3).

**Table 1:** Total DNA yield and number of HSV genomes detected. N.D. = Not Detected.

Samples	N.D. (<1 ng DNA)	1-100 ng DNA	>100 ng DNA	N.D. (<1 viral genome)	1−1,000 viral genomes	>1,000 viral genomes
**Total (2015) n = 229**	41	167	21	47	164	18
**Total (2016) n = 257**	49	164	44	206	47	4
**Total (All) n = 486**	90	331	65	253	211	22

### Sequencing of samples with high HSV genome copy numbers

We selected samples with the highest HSV copy number (Table 1) for our attempts to sequence viral genomes from remote settlements in Namibia. Sequencing libraries were constructed as previously described [29], and samples were subjected to Illumina paired-end deep sequencing. HSV genomes were assembled using an in-house *de novo* viral genome assembly workflow [30]. We successfully recovered viral genome from four samples (Table 2). Other samples attempted for viral genome sequencing yielded insufficient viral sequencing reads for viral genome assembly (Fig 3). The four HSV-1 viral genomes were all derived from oral samples (Table 2). The average coverage depth ranged from >12,000× (sample 038) to 39× (sample 309). Three of these viral genomes (038, 055, and 050) had > 100X coverage across a majority of the genome: HSV1-Namibia-2015-oral038 (12,866 × coverage), HSV1-Namibia-2015-oral055 (722×), and HSV1-Namibia-2015-oral050 (142×). For participant 309, the sequencing libraries of separate oral-external and genital-external libraries both yielded insufficient viral reads to reconstruct independent viral genomes. Instead, we pooled viral sequencing reads across these two samples (23.5k total), which was sufficient to recover a low-coverage (39×) viral genome from this participant: HSV1-Namibia-2016–309combined. To our knowledge, these four viral genomes represent the first HSV-1 genomes derived from Namibia.

**Table 2:** Data and sequencing statistics for samples with successful HSV-1 genome recovery.

Sample #	Sample source	Data collection year	Average coverage depth	Mapped reads	% genome coverage >100×	GenBank Accession #
038	Oral external, adult female	2015	12,866×	7.0 M	99%	PP379031
055	Oral external, juvenile	2015	722×	8.8 M	97%	PP379033
050	Oral external, juvenile	2015	142×	83.5 K	65%	PP379032
309*	Oral external/genital external, adult female	2016	39×	23.5 K	3%	PP379034

* DNA was pooled for two HSV-positive samples (oral-external and genital-external) from study participant 309, to provide sufficient viral DNA templates for oligo-enrichment and sequencing.

### Comparative genomic analysis of Namibian HSV-1 isolates

To place these new viral genomes from Namibia into a global context, we compared their relatedness to previously described HSV-1 genomes from Africa and from all available geographic regions. There are currently only 16 HSV-1 strains from the entire African continent. Fifteen of these were collected in Nairobi, Kenya between 1981−1984 [8] and sequenced almost 30 years later [3]. Additionally, a South African HSV-1 genome collected from a 2008 blood sample was deposited in GenBank [31].

**Fig 4:**
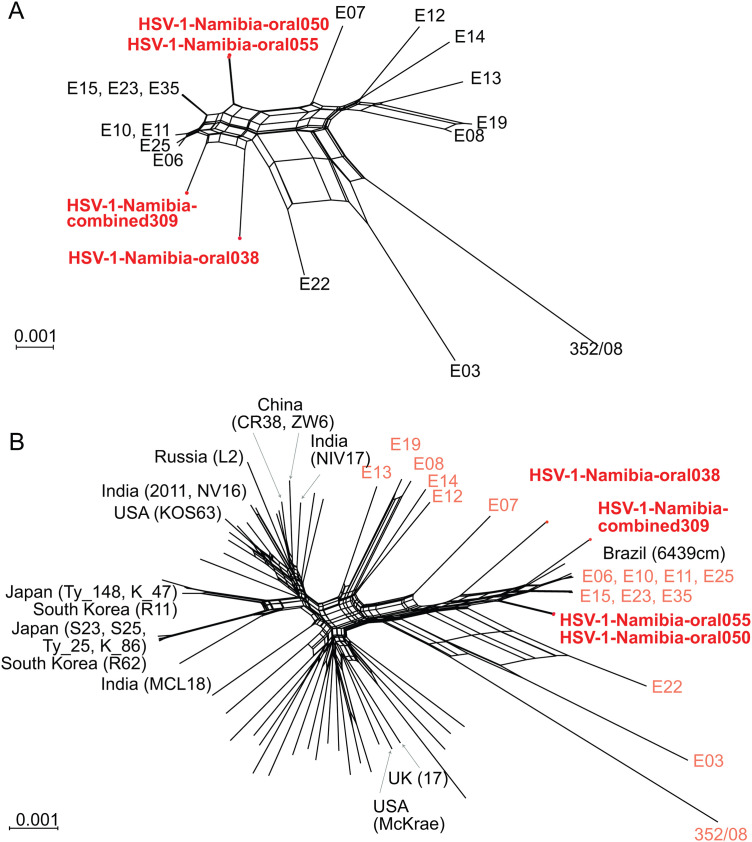
Relatedness between HSV-1 isolates from participant samples and previously collected samples. The four HSV-1 genomes isolated in this study (shown in red) were aligned and compared in (A) a SplitsTree network graph analysis with strains sampled from African nations (B) and a globally representative set of all known HSV-1 genomes. All but one of the African strains were collected in Nairobi, Kenya between 1981−1984, as part of a study by Sakaoka et al [8]. One sample, 352/08, was collected in 2008 in South Africa [31]. In the global network (B), other HSV-1 genomes from African nations are highlighted (pink). Network tips are labeled for viral genomes of Asian origin and for representative strains from the USA (KOS63, McKrae) or Europe (17). The inner reticulations on the network graph indicate signs of past recombination between strains. S1 Table lists the previously sequenced HSV-1 strains used for this comparison.

We first aligned and compared the four uncultured Namibian HSV-1 isolates to these 16 strains from elsewhere in Africa (Fig 4A). We then extended this comparison to a globally representative set of 66 strains (Fig 4B; see S1 Table for list of strains). In both network graph analyses, the four Namibian sequences fit within the scope of viral genetic diversity previously defined by the African strains, which have been noted in prior studies to already encompass a high level of all known HSV-1 genetic diversity [9,10]. The HSV-1 genomes from participants 050 and 055 clustered closely on both network graphs. We also compared the protein-coding and intergenic regions of these four Namibian HSV-1 isolates vs. the set of 66 globally distributed strains. This comparison revealed no variations shared among all four Namibian HSV-1 isolates that distinguished these isolates from others. All coding and non-coding variations in these genomes reflected constellations of those observed in prior African and globally distributed genomes.

### Integrating HSV detection with human contact networks

**Fig 5:**
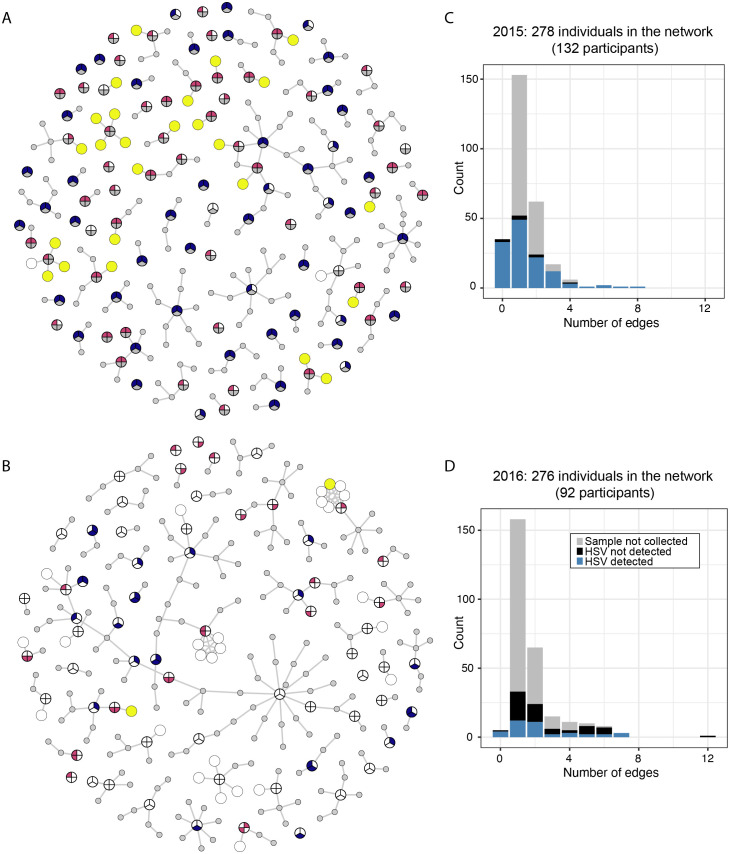
Contact network of study participants. Edges show contacts as reported by participants sampled in (A) 2015 and (B) 2016. The grey nodes represent named contacts who were not sampled for HSV sequencing and did not participate in the study. See Fig 2 for full details of circular sampling-diagrams. If a biological sample was not collected from an individual, the corresponding node is shaded in grey. If HSV was not detected in a sample, the corresponding part of the node is white, and if HSV was detected, the corresponding part of the node is red for female adults, blue for male adults, and yellow for juveniles. The inset bar plots for (C) 2015 (D) and 2016 show the distribution of the number of edges (family ties or sexual contacts) broken down by HSV detection (light blue = HSV detected, dark blue = not detected, grey = not sampled).

Study participants completed interviews at the time of the collection of samples for HSV-detection, providing information on socio-demographics, access to health care, movement and travel, and close interpersonal contacts within and between the settlements in the region [32]. Study participants self-reported social and sexual contacts for the six months preceding the sample collection. In the resulting contact network (see Materials and Methods for details), the study participants and their named contacts comprise the nodes and the edges represent contacts between nodes, including familial contacts and sexual contacts (Fig 5). The network of participants was strongly connected; the majority of the edges linked sexual contacts; 82.3% (153/186) and 75.6% (195/254) in 2015 and 2016, respectively. The family/household ties among the participants made up the remainder of the edges.

We completed a descriptive analysis of the networks focusing on degree distribution, shortest paths, and betweenness centrality. The degree measures the number of individuals to which each individual is linked, or the potential connections along which a transmissible pathogen could spread. We expected shorter paths between infected participants and that infected participants would have a higher betweenness centrality. The average degree distributions in 2015 and 2016 were 1.38 and 1.84, ranging from 0 to 8 and 0–12. The network highlights that sharing sexual partners was not uncommon. We identified sexual partners who participated in the study and were named by one or more study participants. Across both years, the distribution of the length of the shortest paths between participants with HSV shedding detected (from either oral or genital samples) was narrower than it was for participants from whom HSV shedding was not detected. This difference was significant only in 2015. In 2015, the average shortest path between individuals with shedding detected was 2.7 edges, compared to 4.1 edges for individuals for whom shedding was not detected (p = 0.02); in 2016 these distances were 2.3 and 3.5 edges (p = 0.5) (Fig 5, inset graphs). Participants with detected HSV shedding did not have a significantly higher betweenness centrality compared to participants without shedding detected (p = 0.7 and p = 0.7). However, our HSV detection was limited to a single time point and likely captured only asymptomatic shedding because no participants reported symptoms at the time of sample collection. We anticipate that these data underreport the true number of HSV-positive individuals in the population [25]. Future studies that combine these data with seroprevalence detection of past infection may reveal different patterns of degree distribution and betweenness centrality.

## Discussion

We examined HSV shedding in a pastoralist population in the sparsely populated desert region of Kaokoveld, in northwestern Namibia. There are no prior statistics on HSV-1 shedding in this population; these data represent the first measures of the public health burden of this pathogen. Prior studies of sexually transmitted infections (STIs) in this region found a high seroprevalence of HSV-2 and frequent PCR-detection of the bacterial pathogen gonorrhea [24,25,27]. While some study participants self-reported either oral or genital lesions compatible with HSV infection in the year preceding sample collection, none reported active lesions at the time of sample collection. Using self-collected swabs immobilized on FTA cards, we successfully isolated viral DNA from oral and genital swabs, and we successfully recovered viral genomes from four of these samples.

The low technology approach of using FTA card immobilization allowed us to screen a previously under-sampled and hard to reach populations in remote locations (Fig 1) to successfully detect HSV infections and recover HSV genomes. Across two years of sample collection, we found that 48% of samples (233 total) had detectable viral DNA present, but just 4.5% (22 total samples) had 1,000 or more viral genomes detected. This level of viral shedding and detection is not surprising, given a single time-point of sample collection and the lack of symptomatic lesions reported by study participants. The low prevalence of detectable HSV DNA in this study reflect the findings of prior studies on asymptomatic HSV shedding [33–35]. However this level of viral shedding represents the lower threshold of viral genetic material needed for successful genome sequencing, even when using oligonucleotide-bait based enrichment methods [13,14,29,36]. The physical limitation of the starting material likely constrained our recovery capability, resulting in only four viral genomes isolated out of 233 samples with detected viral DNA. Future studies targeting sample collection from symptomatic participants would likely yield a greater number of HSV-positive samples with higher levels of HSV DNA per sample, enabling higher rates of viral genome recovery. However, this study demonstrates that samples collected from asymptomatic individuals in remote populations, residing in harsh environmental conditions can successfully detect HSV infections and recover sequences. This new capability provides avenues to address the current disparity in our knowledge of HSV prevalence and genome sequences from under-sampled regions of the world, including remote and rural populations.

We repeated sample collection at the same two settlements across 2015 and 2016. However, these populations are highly mobile, and these data represent individuals and families from many locations and settlements across Kaokoveld. The area had been experiencing severe drought during our sampling efforts in 2015 (which technically took place during the rainy season), leading to massive cattle die offs and limited food availability. Although our sampling efforts in 2016 occurred during the dry season, there had been sufficient rainfall prior to our arrival, which reduced ongoing herd die-off. More goats were present than the more highly valued, but less drought-resistant, cattle. Subsistence maize gardens had been more productive in 2016 than in 2015. Across samples collected during the drought-phase study in 2015, we detected HSV (>1 genome) in 81% of individuals, but only 20% for 2016. Likewise, there were 18 samples with >1,000 HSV genome copies from 2015, but only 4 such samples in 2016 (Fig 3). It is unclear how environmental stressors, such as drought, might impact HSV shedding. One hypothesis is that drought-induced social and health stress lead to higher rates of viral reactivation and shedding, which would be consistent with our observations from 2015 and 2016 and would require significantly more data collection to evaluate.

Our goal for this study was to add a viral genetic component to a larger, ongoing investigation of the relationships between human movement, access to health care, health vulnerability, and data biases [24–27,32]. We studied remote pastoralist populations in Namibia to address these issues for multiple reasons. First, the location is difficult to access and has limited points of health care. As a result, these populations are largely overlooked in national health population and data from Namibia and globally [37]. Second, the cultural acceptance of extramarital partnerships and polygynous marriage results in high levels of sexual partner concurrency (Fig 5) and transparency in partnerships. We have observed a high prevalence of STIs in this population, including high rates of HSV-2 and gonorrhea, as noted above [24–27]. Finally, a lack of prior data from this area on the prevalence of HSV-1, a common virus, and viral genetic data, highlighted the underrepresentation of these populations in such studies.

If HSV genomes had been recoverable from multiple individuals within the interconnected social and sexual networks of the study participants (Fig 5), it may have been possible to detect past transmission events by measuring current viral relatedness. However, the infrequent and low-level of viral shedding in these asymptomatic participants did not yield sufficient viral genomes to enable this analysis. Future longitudinal studies, involving both symptomatic sample collection and socio-demographic interviews as done here, may provide these insights into human and viral interaction networks. Such studies on STI prevalence and its impacts on rural and remote populations can also inform public health interventions and help illuminate the impact of environmental factors on health.

## Materials and Methods

### Sample acquisition

The study design has approval from Penn State’s Institutional Review Board (IRB #STUDY00001510: Movement and Pathogens in Namibia) and Institutional Biosafety Committee (IBC #48898). We conducted long-form interviews and biological sampling in two settlements in the desert of Kaokoveld, Namibia in 2015 and 2016. Upon arrival in the two Himba settlements, researchers sought and received approval from local settlement authorities at each location to explain the study. We gained their permission and assistance in recruiting volunteers. The sites of data collection were in the Kunene region, east of the Skeleton Coast and northwest of Etosha National Park, just south of the Angolan border. To access these locations, researchers arrived in Windhoek, drove approximately 8 hours to the small town of Opuwo, and then drove approximately 5 hours in a 4x4 vehicle on sand trails to arrive at the settlements where the study was done. The interview data and biological samples exited the study sites with the researchers. The lack of electricity and running water created a need for low-tech sample storage and transport capabilities. All personnel, research equipment, camping necessities, food, and petrol were required to travel in and out of the study settlements, creating space and technology constraints for sample storage and transport.

Each interview and sampling session included exactly one researcher (either Bharti or Hazel) and one voluntary study participant as well as one local resident translator, who read, wrote, spoke, and understood English and spoke and understood the Bantu language Otjiherero, the local language that all study participants spoke. The translator was always the same sex as the study participant; the female translator remained the same across both years, but each male translator held the role for exactly one data collection year. Study participants were restricted to adults. The designation of “adult” was locally determined by household responsibilities, interpersonal relationships, or age when known. In these settlements, individuals are considered adults at approximately 16 years of age. See below for detailed inclusion criteria.

During the rainy season in February 2015, we conducted interviews and collected samples with 102 adults across two Himba settlements. Of these 102 interviews, 25 were part of a pilot phase during which only demographic data were collected with biological samples and full interviews were not conducted and an additional two (2F) provided oral swabs and basic demographic data only as guardians of children. During the dry season in October 2016, we conducted 64 complete interviews paired with biological sample collection with adults across the same two settlements that were studied in 2015. Participants were sampled from the same two Himba settlements each year. Some participants were residents in these settlements and others were visiting when the studies were conducted. Participants were specifically asked if they could recall when they most recently experienced typical symptoms of HSV 1 or 2 infection (oral or genital sores or lesions), if ever.

### Participant interviews

Researchers Bharti and Hazel developed the interview instrument. Interviewers Jakurama and Matundu translated all items from English to Otjiherero, evaluated them for clarity and cultural context, and translated them from Otjiherero back to English. Bharti and Hazel reviewed the back-translations for clarity and adjusted the language with Jakurama and Matundu as necessary to ensure consistent interpretations. Each interview began with an explanation of the interview and sample collection process and purpose of the research, as well as opportunities for each potential participant to ask questions, decide whether to give their verbal consent for study participation, and to decline participation and revoke consent at any point. Enrolled participants were encouraged to ask questions for clarification, were permitted to skip or decline to answer any questions and could decline continuation at any point during the interview.

### Collection of biological samples on FTA cards

Along with each interview conducted with an adult, participants self-swabbed 2–4 tissue sites for this study. Each participant provided verbal consent for swab collection. Participants self-collected swabs of the external oral area including lips and saliva (2015, 2016), and internal oral area of the dorsal tongue (2016) with supervision from a researcher. Participants were then given careful instructions, from a same-sex translator, and privacy behind a curtain, to self-collect swabs of the external genital area (2015 and 2016), and internal genital area (2016, female participants only). Two drops of saline solution without preservatives were added to each swab in a 1.5 mL microcentrifuge tube after sample collection and the wet swab was then rolled onto a Whatman® Flinders Technology Associates (FTA^TM^) card for sample preservation and storage. While the sample on the card was visibly wet, the extent where the sample wetted the card was outlined in pencil with a 2 mm buffer. After the spots dried, cards were closed and stored in sealed envelopes with desiccant packs at ambient temperature (consistently over 40° C during the daytime). After the samples were placed on FTA cards, the swabs themselves were then discarded as biohazardous material. The study used sterile Copan FLOQSwabs®. Unique numeric codes were written on the FTA cards and the paper copies of the data collected during the interviews so they could be paired later for analyses. FTA cards were returned to lab facilities at The Pennsylvania State University for DNA extraction, sequencing, and analysis.

### Study inclusion

Inclusion criteria for participants were as follows: (1) All participants providing a biological sample and completing an interview must be culturally recognized adults who are sexually active. Thus, we recruited participants who were at least 16 years old. We did not have an age maximum for recruitment. (2) The study recruited women in equal proportions to men. Pregnant women were included, as participation posed no risk to them or to the fetus. (3) The population we worked with was ethnically homogenous. All the tribes who live in the areas studied were ethnically, linguistically, or culturally related. (4) Visitors to the area from outside the focal settlements who were likely to be of different ethnic backgrounds were eligible for recruitment and were not excluded. (5) Verbal consent was obtained for all participants. For juveniles, we required verbal consent from a guardian and from the juvenile. Juveniles could then provide a cheek swab with the assistance of their guardian, and their guardian was asked for the names of their immediate family members (parents, siblings). Juveniles were not interviewed and did not provide a genital swab.

### DNA isolation from FTA cards

The area of the card where the sample was located (within the pencil outline) was cut out of the card with a razor blade. Total DNA was extracted from FTA cards using an adapted version of the manufacturer’s protocol [38]. Our specific modifications were that the FTA card pieces were submerged in extraction buffer (10mM Tris-HCl, 10mM EDTA, 10mM NaCl, 2% Sodium dodecyl sulfate in water) and Proteinase K (Fisher), and these were incubated overnight (instead of only 1 hour) at 56° C with agitation. Total DNA was then extracted through multiple rounds of phenol-chloroform extraction, followed by ethanol precipitation. DNA pellets were dried via vacuum centrifuge and resuspended in water. Total DNA was quantified by Qubit® 2.0 Fluorometer (Thermo Fisher). The amount of viral DNA was determined by a previously described quantitative real time PCR (qPCR) assay which amplified a region of the UL27 gene (which encodes glycoprotein B, or gB) and was quantified fluorescently [39,40]. This assay detects the UL27 of both HSV-1 and HSV-2, and it does not distinguish between the two. This quantification served as a measurement of total viral DNA present in each sample. Samples were considered “positive” for HSV if they had detectable viral DNA via qPCR (>1 genome, as compared to a standard curve of cultured HSV-1 DNA). When sufficient card material was available (i.e., based on the outlined area of swab-rolling), repeat DNA extraction and pooling were used to increase yield.

### Next-generation sequencing

Total DNA for each sample to be sequenced was sheared using a Covaris M220 focused-ultrasonicator (Covaris) under the following conditions: 10% duty, power 60, 200 cycles/burst for 60s at 4ºC. Total DNA libraries were then prepared according to manufacturer’s protocols for the KAPA Biosystems HyperPrep Library Kit (with 14 cycles of amplification). Custom HSV-specific oligonucleotide probes [41] were used for in-solution target enrichment, using either Roche Nimblegen SeqCap baits (sample 038) or the Arbor Biosciences myBaits Target Capture Kit (samples 050, 055, 309) according to the manufacturer’s protocols [29,42]. The oligo-enriched library was amplified for 14 cycles using the KAPA HiFi HotStart Library Amplification Kit.

Samples were quantified by Qubit, HSV qPCR, and an adapter-specific qPCR, before multiplexing up to four libraries per flow cell. Libraries were sequenced using an Illumina MiSeq 600-cycle kit with v3 chemistry, according to the manufacturer’s recommendations. Additional Illumina runs for the same source material were concatenated to increase data yield. Prior to genome assembly, a BLAST database consisting of all known HSV-1 and HSV-2 genomes was constructed. This database was used for “positive selection” of HSV-like sequence reads (e-value < 10^−2^). These reads then *de novo* assembled using a published viral genome assembly (VirGA) pipeline for each sample, as previously described [30]. Briefly, after quality control, eight SSAKE *de novo* assemblies were generated and combined into a consensus genome using Celera, GapFiller, and mafnet [43]. Annotations were transferred from the HSV-1 reference genome (strain 17; GenBank accession JN555585) based on homology. GenBank accessions for these 4 genomes are listed in Table 1 ([href:https://www.ncbi.nlm.nih.gov/nuccore/PP379031,PP379032,PP379033,PP379034]PP379031-PP379034). Raw sequence data are available via the Sequence Read Archive (SRA) under BioProject [href:https://www.ncbi.nlm.nih.gov/bioproject/PRJNA1347855]PRJNA1347855 (SRA accessions SAMN52875394-SAMN52875397).

### Viral genome network graph analysis

We obtained four new HSV-1 genomes from this study. We aligned these four sequences with other HSV-1 genomes using ClustalW2 [44]. S1 Table contains the full list of previously published HSV-1 genomes used for these comparisons, along with their geographic source, GenBank accession, and reference(s). Multiple Alignment using Fast Fourier Transform (MAFFT) was used to construct pairwise global nucleotide alignments between trimmed genome sequences (i.e., terminal repeats were removed to avoid duplicated influence on the network graph). The Africa-specific alignment contains 132,024 ungapped bases (139,031 total in alignment), while the global alignment contains 122,753 ungapped bases (163,982 total in alignment). The MAFFT alignments were used to generate NeighborNet network graphs in SplitsTree4, using the uncorrected P distance and excluding gaps [45].

### Contact network among study participants

Using the contact information provided during the long-form interviews, we constructed a contact network for each year of data collection that included household members, family members, and sexual contacts of the study participants. The contact information on family and sexual partners provided links between participants as well as individuals who did not participate in the study (Fig 5); study participants and their named contacts comprise the nodes, edges indicated contact between nodes. Participants’ named sexual partners in the three months preceding data collection appear in the network as additional vertices and provided a more complete picture of the connectivity in the study population. Named contacts who did not participate in the study were not located or screened for HSV shedding. We used detectable HSV DNA (> 1 genome by qPCR) as an indicator of HSV infection for the contact-network analysis.

## Supporting information

S1 Table Table of sources of HSV-1 genomes for network graph analysis. (PDF)
