# Supplementary material for: Herpes simplex virus detection and genomes from under-sampled, remote populations
Source: PLoS One. 2026 Jul 24;21(7):e0344138. doi: 10.1371/journal.pone.0344138 (PMC13399521; doi:10.1371/journal.pone.0344138)
Supplement: S1 Table — (PDF) [file pone.0344138.s001.pdf]

Supplemental Table 1: Sources of HSV-1 genomes for network graph analysis

Virus Isolate	Country (with location detail, if available)	GenBank Accession #	Genome Reference(s)
17 (HSV-1 Reference genome)	U.K. (Glasgow)	JN555585	[1]
E03	Kenya (Nairobi)	HM585509	[1]
E06	Kenya (Nairobi)	HM585496	[1]
E07	Kenya (Nairobi)	HM585497	[1]
E08	Kenya (Nairobi)	HM585498	[1]
E10	Kenya (Nairobi)	HM585499	[1]
E11	Kenya (Nairobi)	HM585500	[1]
E12	Kenya (Nairobi)	HM585501	[1]
E13	Kenya (Nairobi)	HM585502	[1]
E14	Kenya (Nairobi)	HM585510	[1]
E15	Kenya (Nairobi)	HM585503	[1]
E19	Kenya (Nairobi)	HM585511	[1]
E22	Kenya (Nairobi)	HM585504	[1]
E23	Kenya (Nairobi)	HM585505	[1]
E25	Kenya (Nairobi)	HM585506	[1]
E35	Kenya (Nairobi)	HM585507	[1]
R11	South Korea (Seoul)	HM585514	[1]
R62	South Korea (Seoul)	HM585515	[1]
S23	Sapporo, Japan	HM585512	[1]
S25	Sapporo, Japan	HM585513	[1]
CR38	China (Shenyang)	HM585508	[1]
Ty_25	Japan	MH999840	N/A
Ty_148	Japan	MH999841	N/A
K_86	Japan	MH999839	N/A
K_47	Japan	MH999838	N/A
ZW6	China	KX424525	N/A
HSV-1/0116209/India/2011	India	KJ847330	[2]
MCL-18-H-1091	India	MH480513	N/A
NIV1613762	India	MH319852	[3]
NIV1722974	India	MG646679	[3]
6439cm	Brazil	MT876428	[4]
352/08	South Africa	OQ102003	N/A
McKrae	Gainesville, FL	JQ730035, JX142173	[5–7]
KOS63	U.S.A. (Houston, TX)	KT425110	[8]
KOS79	U.S.A. (Madison, WI)	KT425109	[8]
CJ994	U.S.A. (Madison, WI)	KR011283	[9]
RE	U.S.A. (New Orleans, LA)	KF498959	N/A
OD4	U.S.A. (Madison, WI)	JN420342	[10]
H129	San Francisco, CA	GU734772	[11,12]
F	Chicago, IL	GU734771	[11,13]
H166	U.S.A.	KM222726	[14]
H166syn	U.S.A.	KM222727	[14]
H193	U.S.A.	KT425108	N/A
HF10	New York, NY	DQ889502	[15]

Virus Isolate	Country (with location detail, if available)	GenBank Accession #	Genome Reference(s)
H1211_F-11	Finland	MH999843	[16,17]
H1215_M-15	Finland	MH999846	[16,17]
H12113_ F-13	Finland	MH999842	[17]
H12114_F-14g	Finland	MH999844	[16,17]
H12117_F-17	Finland	MH999845	[16,17]
H12118_F-18g	Finland	MH999847	[16,17]
H1311_F11*l*	Finland	MH999848	[17]
H1312_M-12	Finland	MH999849	[17]
H1412_F-12g	Finland	MH999851	[17]
H15119_M-19	Finland	MH999850	[17]
SC16	Spain (Madrid)	KX946970	[18]
172_2010	Jena, Germany	LT594105	[19]
2158_2007	Jena, Germany	LT594106	[19]
3083_2008	Jena, Germany	LT594107	[19]
1319_2005	Germany	LT594108	[19]
270_2007	Manebach, Germany	LT594109	[19]
66_2007	Jena, Germany	LT594110	[19]
1394_2005	Germany	LT594111	[19]
369_2007	Jena, Germany	LT594112	[19]
160_1982	Erfurt, Germany	LT594192	[19]
132_1998	Gelsenkirchen, Germany	LT594457	[19]
L2	Russia (Moscow)	KT780616	[20]

References

1. Szpara ML, Gatherer D, Ochoa A, Greenbaum B, Dolan A, Bowden RJ, et al. Evolution and diversity in human herpes simplex virus genomes. J Virol. 2014;88: 1209–27. doi:10.1128/JVI.01987-13

2. Bondre VP, Sankararaman V, Andhare V, Tupekar M, Sapkal GN. Genetic characterization of human herpesvirus type 1: Full-length genome sequence of strain obtained from an encephalitis case from India. Indian J Med Res. 2016;144: 750–760. doi:10.4103/ijmr.IJMR_747_14

3. Nyayanit DA, Sahay RR, Sakpal GN, Shete AM, Chaubal GC, Sarkale P, et al. Identification and phylogenetic analysis of herpes simplex virus-1 from clinical isolates in India. Access Microbiol. 2019;1. doi:10.1099/acmi.0.000047

4. Hernandez JM, Singam H, Babu A, Aslam S, Lakshmi S. SARS-CoV-2 Infection (COVID-19) and Herpes Simplex Virus-1 Conjunctivitis: Concurrent Viral Infections or a Cause-Effect Result? Cureus. 2021. doi:10.7759/cureus.12592

5. Watson G, Xu W, Reed A, Babra B, Putman T, Wick E, et al. Sequence and comparative analysis of the genome of HSV-1 strain McKrae. Virology. 2012;433: 528–37. doi:10.1016/j.virol.2012.08.043

6. Macdonald SJ, Mostafa HH, Morrison LA, Davido DJ. Genome sequence of herpes simplex virus 1 strain McKrae. J Virol. 2012;86: 9540–9541. doi:10.1128/JVI.01469-12

7. Williams LE, Nesburn AB, Kaufman HE. Experimental induction of disciform keratitis. Arch Ophthalmol. 1965;73: 112–114. doi:10.1001/archopht.1965.00970030114023

8. Bowen CD, Renner DW, Shreve JT, Tafuri Y, Payne KM, Dix RD, et al. Viral forensic genomics reveals the relatedness of classic herpes simplex virus strains KOS, KOS63, and KOS79. Virology. 2016;492: 179–186. doi:10.1016/j.virol.2016.02.013

9. Lee K, Kolb AW, Sverchkov Y, Cuellar JA, Craven M, Brandt CR. Recombination Analysis of Herpes Simplex Virus 1 Reveals a Bias toward GC Content and the Inverted Repeat Regions. Longnecker RM, editor. J Virol. 2015;89: 7214–7223. doi:10.1128/JVI.00880-15

10. Kolb AW, Adams M, Cabot EL, Craven M, Brandt CR. Multiplex sequencing of seven ocular herpes simplex virus type-1 genomes: phylogeny, sequence variability, and SNP distribution. Invest Ophthalmol Vis Sci. 2011;52: 9061–73. doi:10.1167/iovs.11-7812

11. Szpara ML, Parsons L, Enquist LW. Sequence variability in clinical and laboratory isolates of herpes simplex virus 1 reveals new mutations. J Virol. 2010;84: 5303–13. doi:10.1128/JVI.00312-10

12. Dix RD, McKendall RR, Baringer JR. Comparative neurovirulence of herpes simplex virus type 1 strains after peripheral or intracerebral inoculation of BALB/c mice. Infect Immun. 1983;40: 103–112. doi:10.1128/iai.40.1.103-112.1983

13. Ejercito PM, Kieff ED, Roizman B. Characterization of herpes simplex virus strains differing in their effects on social behaviour of infected cells. J Gen Virol. 1968;2: 357–364.

14. Parsons LR, Tafuri YR, Shreve JT, Bowen CD, Shipley MM, Enquist LW, et al. Rapid Genome Assembly and Comparison Decode Intrastrain Variation in Human Alphaherpesviruses. mBio. 2015;6: e02213-14. doi:10.1128/mBio.02213-14

15. Ushijima Y, Luo C, Goshima F, Yamauchi Y, Kimura H, Nishiyama Y. Determination and analysis of the DNA sequence of highly attenuated herpes simplex virus type 1 mutant HF10, a potential oncolytic virus. Microbes Infect. 2007;9: 142–149. doi:10.1016/j.micinf.2006.10.019

16. Paavilainen H, Lehtinen J, Romanovskaya A, Nygårdas M, Bamford DH, Poranen MM, et al. Inhibition of clinical pathogenic herpes simplex virus 1 strains with enzymatically created siRNA pools. J Med Virol. 2016;88: 2196–2205. doi:10.1002/jmv.24578

17. Bowen CD, Paavilainen H, Renner DW, Palomäki J, Lehtinen J, Vuorinen T, et al. Comparison of herpes simplex virus 1 strains circulating in Finland demonstrates the uncoupling of whole-genome relatedness and phenotypic outcomes of viral infection. Longnecker RM, editor. J Virol. 2019;93: e01824-18. doi:10.1128/JVI.01824-18

18. Rastrojo A, López-Muñoz AD, Alcamí A. Genome Sequence of Herpes Simplex Virus 1 Strain SC16. Genome Announc. 2017;5: e01392-16. doi:10.1128/genomeA.01392-16

19. Pfaff F, Groth M, Sauerbrei A, Zell R. Genotyping of herpes simplex virus type 1 (HSV-1) by whole genome sequencing. J Gen Virol. 2016. doi:10.1099/jgv.0.000589

20. Skoblov MYu, Lavrov AV, Bragin AG, Zubtsov DA, Andronova VL, Galegov GA, et al. The genome nucleotide sequence of herpes simplex virus 1 strain L2. Russ J Bioorganic Chem. 2017;43: 140–142. doi:10.1134/S1068162016060133
